# Supervised contrastive loss helps uncover more robust features for photoacoustic prostate cancer identification

**DOI:** 10.3389/fonc.2025.1592815

**Published:** 2025-07-09

**Authors:** Yingna Chen, Feifan Li, Zhuoheng Dai, Ying Liu, Shengsong Huang, Qian Cheng

**Affiliations:** ^1^ School of Information Engineering, College of Science & Technology Ningbo University, Ningbo, Zhejiang, China; ^2^ School of Physics Science and Engineering, Tongji University, Shanghai, China; ^3^ Department of Urology, Tongji Hospital, Tongji University School of Medicine, Shanghai, China; ^4^ The National Key Laboratory of Autonomous Intelligent Unmanned Systems, China, The Frontiers Science Center for Intelligent Autonomous Systems, Ministry of Education, Shanghai, China

**Keywords:** supervised contrastive learning, photoacoustic spectral analysis, prostate cancer, robust feature, CNN

## Abstract

**Background:**

Photoacoustic spectral analysis has been demonstrated to be efficacious in the diagnosis of prostate cancer (PCa). With the incorporation of deep learning, its discrimination accuracy is progressively enhancing. Nevertheless, individual heterogeneity persists as a significant factor that impacts discrimination performance.

**Objective:**

Extracting more reliable features from intricate biological tissue and augmenting discrimination accuracy of the prostate cancer.

**Methods:**

Supervised contrastive learning is introduced to explore its performance in photoacoustic spectral feature extraction. Three distinct models, namely the CNN-based model, the supervised contrastive (SC) model, and the supervised contrastive loss adjust (SCL-adjust) model, have been compared, along with traditional feature extraction and machine learning-based methods.

**Results:**

The outcomes have indicated that the SCL-adjust model exhibits the optimal performance, its accuracy rate has increased by more than 10% compared with the traditional method. Besides, the features extracted from this model are more resilient, regardless of the presence of uniform or Gaussian noise and model transfer. Compared with CNN model, the transfer performance of the proposed model has improved by approximately 5%.

**Conclusions:**

Supervised contrast learning is integrated into photoacoustic spectrum analysis and its effectiveness is verified. A comprehensive analysis is conducted on the performance improvement of the proposed SCL-adjust model in photoacoustic prostate cancer diagnosis, its resistance to noise, and its adaptability to the data heterogeneity of different systems.

## 1 Introduction

According to the 2023 cancer statistics, PCa alone accounts for 29% of all incident cases, signifying that it has the highest number of diagnoses and incurs the largest number of deaths among men (1). In spite of the existence of numerous diagnostic modalities such as tissue biopsy, digital rectal examination (DRE), prostate-specific antigen (PSA) detection, transrectal ultrasound imaging (TRUS), and magnetic resonance imaging (MRI), the rates of false positives and false negatives remain relatively high (**Table 1**) (2–4). To meet the escalating demand for diagnostic techniques that are more accurate and less invasive, recent years have witnessed remarkable progress in novel detecting methods.

**Table 1:** Overview of various diagnostic modalities for PCa (4).

Modality	Sensitivity (%)	Specificity (%)	Advantages	Disadvantages
DRE	37	20	Simple and easy to operate	Cannot be used for early detection; low reliability.
PSA test	72.1	93.2	Prostate tissue-specific.	Not specific to PCa.
TRUS-guided biopsy	66.1	96	Golden standard	Invasive; discomfort and the potential for infection
MRI (DWI)	69	89	Its applications encompass the domains of PCa	Poor real-time performance
MRI (T2WI)	60	76		
MRI (mpMRI)	93	41		Relatively higher rate of false- positive results
PAI	81.3	96.2	Non-ionizing;non-invasive;real-time	Limited deep tissue penetration

Photoacoustics combines the capacity of light absorption spectroscopy to distinctly identify biomolecules with the proficiency of ultrasound detection to withstand scattering by biological tissues. Its applications within the realm of biomedicine have been comprehensively investigated and substantiated (5, 6), as well as in the context of prostate cancer (7–9). Nevertheless, the majority of current experiments are founded on imaging intensity, which hinges on the absorption of tissues at particular optical wavelengths, thereby constricting the volume of acquired information (10–13). Additionally, imaging intensity is highly susceptible to individual variances, rendering the attainment of a more accurate quantitative assessment arduous.

Studies have demonstrated that multi-wavelength photoacoustic spectroscopy facilitates the enhanced detection of alterations in tissue chemical constituents and the progression of heterogeneity (14–17). Our prior research also established that multi-wavelength photoacoustic spectroscopy is efficacious in identifying the prostate, whether in punctured tissue strips or intact ex vivo tissues (18, 19). However, the high dimensionality of multi-wavelength photoacoustic spectroscopy poses a challenge in feature extraction. Fortunately, the swift advancement of deep learning has opened up novel prospects for this method. Although the majority of artificial intelligence (AI)-assisted endeavors primarily concentrate on the optimization of photoacoustic imaging (11, 20), in recent years, it has been progressively applied to photoacoustic spectrum analysis, especially for feature extraction and classification as summarized in **Table 2** (14, 18, 21–27). It is evident that machine learning, inclusive of deep learning, is still in a rather nascent stage in photoacoustic spectrum analysis. The majority of work still depends on hand-crafted features and traditional machine learning. One of the principal factors could be the paucity of data sets, which restricts the utilization of more intricate and advanced deep learning models.

**Table 2:** Machine learning used for various biomedical applications in photoacoustic spectrum analysis.

Stages of application	Objective	Architecture & algorithm	Reference
Parameter inversing	Fitted the PA signal spectrum and the adipocyte size	Deep Neural Network with Fully Connected Layers	(21)
	Photoacoustic spectral unmixing	Autoencoders	(22)
	Semi-quantify bone mineral density	Fully connected multi-layer deep neural network	(23)
Feature extraction and prostate cancer diagnosis	Dimension reduction and classification	Linear Discriminant Analysis (LDA), Quadratic Discriminant Analysis (QDA), ResNet-18	(18)
Feature extraction for breast cancer subtype identification	Feature wavelengths selection	Partial least-squares discriminant algorithm (PLS–DA)	(14)
Glucose concentration prediction	Dimension reduction and fitting the glucose concentration	Linear Regression (LR), Support Vector Regression (SVR), Random Forest Regression (RFR), Adaboost, LightGBM, Artificial Neural Network (ANN) and Gaussian Process Regression (GPR)	(24)
Assessment of Breast Tumor Progression	Dimension reduction and classification	SVM, SVM- Radial Basis Function (SVM-RBF), SVM-Polynomial, SVM- Linear, KNN, PLSDA & SVMDA	(25–27)

Consequently, we selected a classical deep learning model to validate its effectiveness in photoacoustic spectrum analysis and prostate cancer diagnosis. In addition, contrastive learning was incorporated to mitigate the impact of inter- and intra-patient variability.

Contrastive learning approaches have exhibited remarkable potential in extracting robust features, particularly in the unsupervised domain (28–31). The central tenet of contrastive learning is to augment the robustness of extracted samples by constructing positive and negative sample pairs. This methodology strives to reduce the intra-class distance among positive pairs and expand the inter-class distance between negative pairs. Nevertheless, the performance of unsupervised contrastive learning models remains inferior to that of supervised learning. Therefore, our focus turns to supervised contrastive learning methods. This approach capitalizes on label information from known samples (training samples) to preclude the generation of incorrect sample pairs that might otherwise affect training results, thereby fortifying the robustness of the extracted features (32, 33).

Finally, the data from diverse systems was also examined to evaluate the model’s generalization performance. These three aspects, namely deep learning for feature extraction, patient variation, and system influence, are all crucial for prostate cancer diagnosis. Based on our research, no prior study has jointly analyzed these three aspects within the context of photoacoustic spectrum analysis. Beyond the introduction, this study is structured into four parts: the Methods section, where the dataset is introduced and an analysis of the employed algorithms and specific models, such as photoacoustic spectral analysis and data preprocessing, is provided; the Experimental section, which presents detailed experimental outcomes; the Discussion section, in which the details of the methods and their limitations are deliberated; and the Conclusion section, which offers a summary of the paper.

## 2 Materials and methods

### 2.1 Data collection and ethical approval

**Figure 1:**
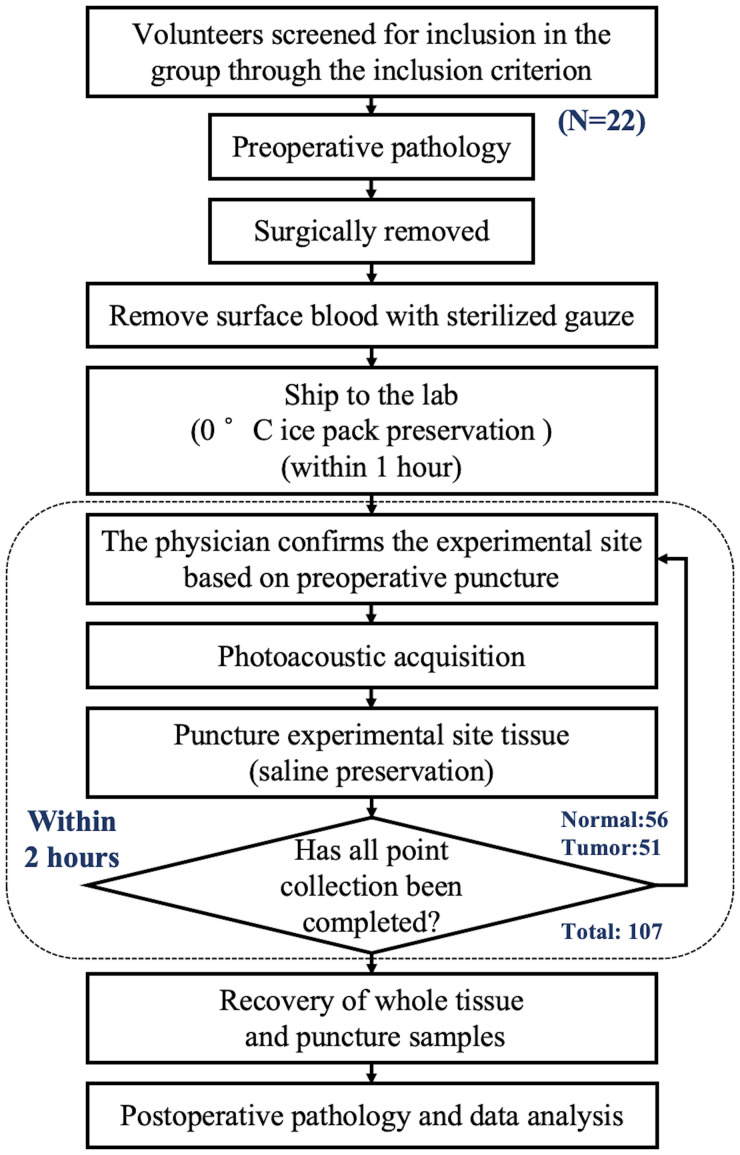
Diagram of sample entry and collection process.Diagram of sample entry and collection process.

This study was carried out in cooperation with Tongji Hospital. The Institutional Review Committee of Tongji Hospital gave its approval for the project experiments. Volunteers were recruited after signing an informed consent form and underwent screening to ensure that they had not received any previous treatment and were suitable candidates for surgical resection. A flowchart of the experiment is presented in **Figure 1**.

The first system collected data from 12 volunteers, while the second system collected data from 10 volunteers. The patients were aged between 61 and 85 years, with an average age of 71.9 years. The overall acquisition structures of the two systems were the same, except for the calibration system. The data collected by the two systems could be used for model validation and testing respectively, thereby ensuring the robustness of the model. Signals were obtained from multiple sites under the guidance of preoperative pathology. To further ensure the pathological characteristics of the photoacoustic detection sites, each site underwent an additional puncture at the same location after detection, and the samples were sent back to the hospital for pathological analysis at the end of the experiment. This process was used to determine the histopathological characteristics of the detected sites and serve as labels for the detected tissues.

It was indeed found in the experiment that there were sometimes discrepancies between the pre-experimental and post-experimental pathological results. However, due to the diffusive nature of prostate cancer, pre-experimental (or preoperative) puncture provides only a rough localization, which may deviate from the photoacoustic detection sites. Therefore, the post-experimental pathological results were uniformly adopted as the labels for the samples.

The photoacoustic signal acquisition system employed in the experiment has been described in detail in our previously published work (18) and thus will not be reiterated herein. In the experiment, photoacoustic signals were collected at 77 wavelengths (ranging from 690 to 950 nm and from 1200 to 1690 nm, with a wavelength increment of Δλ = 10 nm) for each point. The collected multi-wavelength signals were then subjected to power spectrum calculation using the subfunction pwelch in MATLAB 2019B. The default Hamming window was utilized as the window function, which had a length of 2500 sampling points and an overlap rate of 90%. The spectral resolution was 0.1 MHz, and the sampling rate was 250 MHz. After the transducer frequency response correction and the wavelength energy correction of the blackbody, the multi-wavelength photoacoustic power spectrum within the range of 1 to 10 MHz was obtained. The data were stored in a 77 × 90 matrix, which served as the initial high-dimensional data for photoacoustic spectral feature extraction. Considering the number of samples, the format of the constructed raw dataset was *N*× 77 × 90, where *N* represented the number of samples (in this paper, *N* is 107), and 77 × 90 denoted the multi-wavelength spectral features.

Both our previous studies and those of other researchers have demonstrated that the linear fitting of photoacoustic spectra in the middle and high frequencies can mirror the variation in heterogeneity within the tissue. Based on this, three parameters, namely the slope, intercept, and median of the linear fit within the 1–10 MHz range of the spectrum, were extracted. Considering that these parameters are non - independent, we focused only on the slope and median and conducted the statistical analysis.

### 2.2 Network architectures

**Figure 2:**
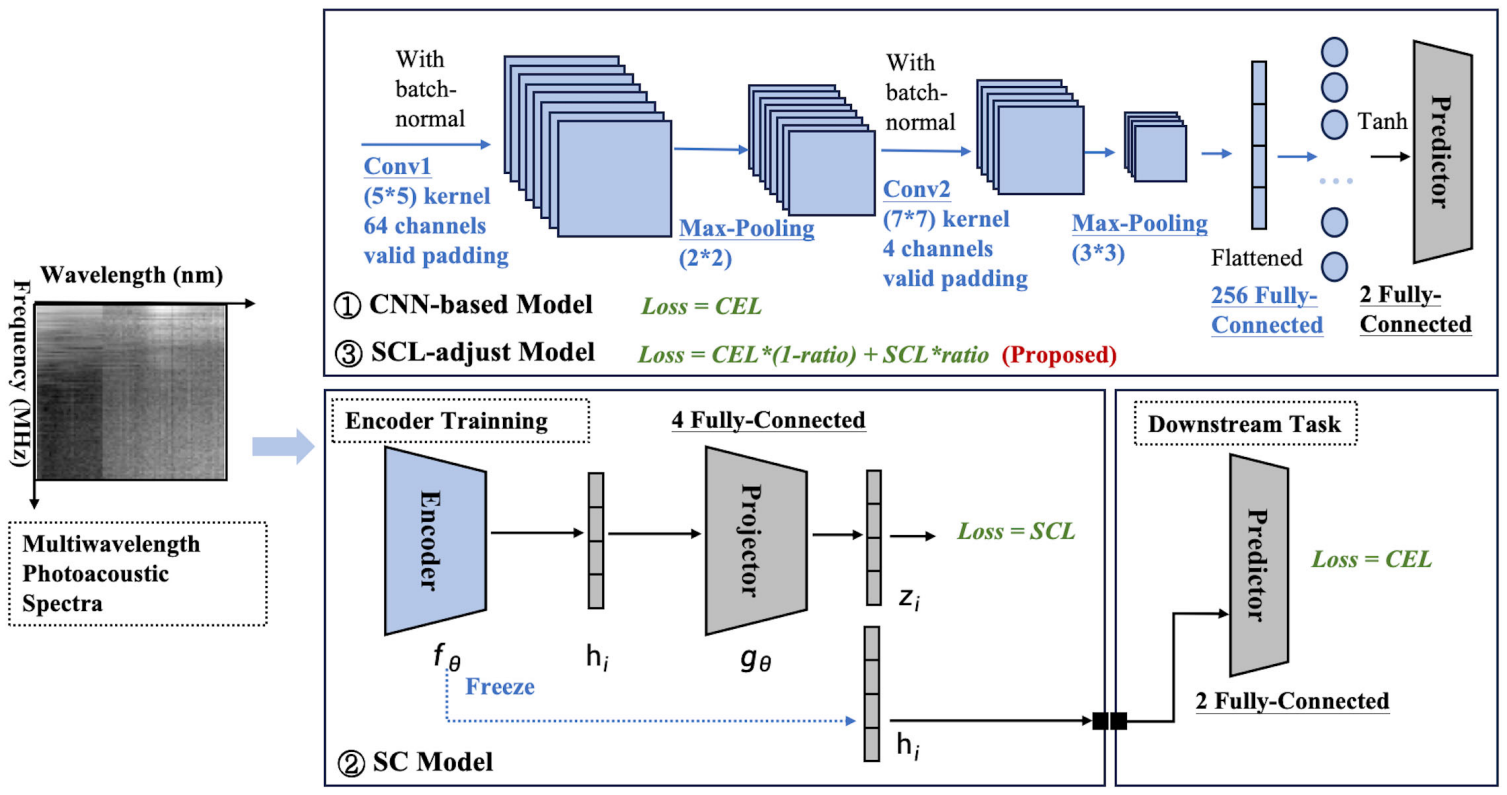
Model framework diagram. Three modeling frameworks are involved: the CNN-based model, SC model, and SCL-adjust model. Blocks with thesame color indicate that they share the same architecture and hyperparameters.

CNNs are widely recognized as a potent feature extraction model for spatial distribution data. Our prior research also indicated that the spatial features of photoacoustic spectra are associated with disease progression. Owing to the restricted number of samples, the constructed network ought not to be overly complex in depth. The optimal architecture of the CNN-based baseline model and its hyperparameters were ascertained through ablation studies and random searches in Section 3B. The detailed basic CNN model architecture can also be observed in **Figure 2** in blue. Cross-entropy is used as the loss function (CEL).

Based on the optimized baseline model, two contrastive models were constructed (**Figure 2**). The first one was a SC model, which comprised an encoder and a projector. The encoder of this model employs exactly the same structure as the baseline CNN (indicated by the same color). Serving as the feature extractor in the contrastive learning model, it is denoted as $f_{\theta }$ . The output feature $h_{i}$ (a 256-dimensional vector here) represents the input representation of the model and can be used for downstream tasks. To enhance the effectiveness of the representation, before performing downstream tasks, $h_{i}$ is mapped to a latent space using a projector (projection head), denoted as $g_{\theta }$ in the **Figure 2** and the mapped feature is denoted as $z_{i}$ . The supervised contrastive learning loss [SCL, defined in Equations 1 and 2 (34)] is applied to $z_{i}$ . This loss function is designed to pull similar samples closer together in the feature space while pushing dissimilar samples apart, thereby enhancing the discriminative power of the learned representations.

The dimensionality selection of the projector significantly impacts model performance. From an information-theoretic perspective (35), it is proven that the projector dimension must satisfy $D\geq \log_{2}C$ (for binary classification, C=2→D≥1 ). Generally, higher dimensions lead to stronger separability in the feature space but require more data for support. Therefore, in this paper, the projector dimension is also treated as a tunable hyperparameter. The optimal parameter selected via the random parameter method is 4, which aligns well with the criteria for selecting the projector dimension.

The above process is collectively referred to as representation learning. Subsequently, the projector is removed, and the downstream task training is performed. Here, the classifier maintains the same dimension as the baseline CNN classifier, and CEL is also used as the loss function. Each layer of the SC is labeled, and **Table 3** summarizes the number of kernels and the size of the feature maps’ output at each layer.

**Table 3:** Summary of data at each layer of the SC model.

Layer		Number of kernels/feature maps	Size of output feature maps
PA spectrum	-	-	77×90
Encoder	Conv1	64	64×77×90
	Max-pooling1	2×2	64×38×45
	Conv2	4	4×38×45
	Max-pooling2	3×3	4×12×15
	Fully-connected layer	256	256
Projector	Fully-connected layer	4	4
Prediction head	Fully-connected layer	2	2


(1) $$\begin{array}{c} s_{i,j}=\frac{z_{i}^{T}z_{j}}{∥z_{i}∥\;∥z_{j}∥} \end{array}$$

Where i,j,k denote the ordinal numbers of the samples; $z_{j}$ is the feature vector of sample *j*, which is the output of the projector; the notation $z_{i}^{T}$ represents the transpose of the feature vector $z_{i}$ . This transposition is used to compute the dot product. $∥z_{i}∥$ represents the norm (or magnitude) of the feature vector $z_{i}$ . $s_{i,j}$ is the cosine similarity of the feature vectors of samples *i* and *j*; It’s used to measure the similarity between feature vectors of samples.


(2) $$\begin{array}{c} SCL=-\sum_{i=1}^{M}\frac{1}{M_{y_{i}}-1}\sum_{j=1}^{M}l_{i\neq j}l_{y_{i}=y_{j}}\text{ln}[\frac{\exp(\frac{s_{i,j}}{t})}{\text{exp}(\frac{s_{i,j}}{t})+\sum_{k=1}^{M}l_{y_{i}\neq y_{k}}\exp(\frac{s_{i,k}}{t})}] \end{array}$$

Where *M* represents the batch size; *y_i_* is the label of the corresponding sample *i*; $M_{yi_{\;}}$ is the sample size in a batch labeled *y_i_*; $l_{i\neq j}\in \{0,1\}$ is 1 when i≠j , and 0 otherwise. It’s used to select different sample pairs; $l_{y_{i}=y_{j}}$ functions similarly which is used to filter same-label sample pairs; on the other hand, $l_{y_{i}\neq y_{k}}$ is used to filter sample pairs with different labels. $s_{i,j}$ and $s_{i,k}$ is the cosine similarity as shown in Equation 1 and *t* is a hyper-parameter. Generally, the smaller *t*, challenging it becomes for the model to concentrate on samples that are difficult to distinguish (36). In this study, we set it to 0.5 to achieve a balance.

The other was a SCL-adjust model, which modified the loss of the basic CNN model. Its architecture is completely identical to the CNN model, with the only modification being the addition of SCL to the model’s loss function. To prevent weight imbalance, the loss function of the SCL-adjust model is defined as SCL*ratio+CEL*(1−ratio) , where ratio represents the weighting coefficient between the two losses. To determine the optimal weight ratio, the proportion of the two losses is optimized using a random parameter search method.

### 2.3 Training

The experiments were conducted using Python 3.8 on a Windows 10 system with an NVIDIA RTX 3060 GPU. Key dependencies include PyTorch 2.4. The random seed was set to 42 for reproducibility. The code used to produce the results reported in this article is available in the supplementary.

The data collected by the first system which contains 12 data groups of volunteers is initially used for model training, and the same model is then applied to the other set of data. To ensure that individual data is not leaked and to account for individual variations, the article does not randomly shuffle all data but rather focuses on the data from individual volunteers. Due to differences in samples from different volunteers, the number of acquisition points in the experiment also varies. Twelve patients were randomly shuffled, and 75% of the volunteer sample data (with the number of selected volunteers being an integer) was extracted as the training set, i.e., 9 patients, with the remaining samples as the test set. In the training set, samples from another 2 patients (approximately 20%) were randomly selected as the validation set. **Table 4** provides a detailed display of the number of sample points and labels in the training set, validation set, and test set of both two datasets. According to the data in **Table 4**, a total of 57 sample points were collected from 12 volunteers, including 37 in the training set, and 10 each in the test set and validation set. In terms of sample points, the training set accounts for approximately 65%, while the test set and validation set each account for about 17.5%, which represents a reasonable proportion distribution. From the perspective of the distribution of normal and tumor samples, the dataset contains more normal data (approximately 60%), which is a common phenomenon in the experiment. This leads to an imbalance in the training set, where normal samples outnumber tumor samples. Fortunately, the proportions of tumor samples in the test set and validation set are relatively balanced. This helps prevent the model from being biased toward normal samples and ensures the reliability of the model. Since the post-training data group does not require a validation set, only the data from three volunteers are divided into test sets, with the rest being allocated to the training sets.

**Table 4:** Distribution of sample points by volunteers in each dataset.

Datasets	Normal (Label = 0)	Tumor (Label = 1)	Datasets	Normal (Label = 0)	Tumor (Label = 1)
Training set	3	3	Validation set	3	2
	5	0		1	3
	5	0	Test set	0	4
	1	4		2	1
	6	0		1	2
	3	2			
	4	1			

All models in this paper are optimized using Stochastic Gradient Descent (SGD). Three models were trained with different loss functions: The CNN model was constrained by the conventional CEL for classification tasks. The SC-model first underwent representation learning using the SCL with an encoder and a projector; subsequently, the trained encoder was combined with a predictor for downstream classification tasks, with the CEL employed as the loss function. The SCL-adjust model, based on the CNN architecture, used a weighted sum of the supervised contrastive loss (SCL) and cross-entropy loss (CEL) as the loss function. The loss weight ratio of SCL-adjust model as well as the momentum of SGD is treated as hyperparameters and optimized via random parameter selection. The learning rate and batch size, as hyperparameters, were also determined using the same method through the validation set. For each model, 500 rounds of random parameter experiments were conducted, and the parameter combination with the best performance on the validation set was selected. Each model was trained for 20 epochs.

### 2.4 Evaluation matrix

Five evaluation metrics are put forward to quantitatively assess the performance of the proposed method. A confusion matrix, which encompasses True Positives (TP), True Negatives (TN), False Positives (FP), and False Negatives (FN), is commonly utilized for representing classification quality. Subsequently, a variety of criteria can be computed based on these four numbers. For specific biomedical problems, it is more crucial to concentrate on the criteria that take into account the number of cases that are actually healthy but are predicted as cancerous (i.e., False Positives), as this may lead to misdiagnosis. Of course, the criteria considering the number of cases that are actually cancerous but are predicted as healthy are also of significant importance, since this may result in missed diagnoses and delayed treatment. Ultimately, in accordance with the literature, in this paper, aside from accuracy, we use precision, recall, specificity, and the Area Under the Curve (AUC) as follows and score is the mean value of these five parameters.

1) The *accuracy* is denoted by the proportion of correct classifications, which gives a general prediction performance of the model, as shown in Equation 3:


(3) $$accurcay=\frac{TP+TN}{TP+TN+FP+FN}$$

2) In the case of binary cancer diagnosis, the *precision*, also called positive predictive value, is defined as the proportion of the correctly predicted cancerous samples in all the actual cancerous samples which is inversely proportional to the misdiagnose rate, as shown in Equation 4:


(4) $$precision=\frac{TP}{TP+FP}$$

3) The *recall*, also called sensitivity, is defined as the proportion of the correctly predicted cancerous samples in all the predicted cancerous sample, which is used to evaluate the ability to recognize positive samples of the model, as shown in Equation 5. It’s inversely proportional to the missed diagnose rate.


(5) $$recall=\frac{TP}{TP+FN}$$

4) The *specificity*, also known as the harmonic mean of the precision and the recall, as shown in Equation 6. This matric conveys the balance between the precision and the recall and reaches its best value at 1 and worst at 0.


(6) $$specificity=\frac{TN}{TN+FP}$$

5) *AUC*, which is defined as the area under receive operating characteristic (ROC) curve, as shown in Equation 7, gives a quantitative evaluation of models avoiding the influence of sample distribution and unbalance. When its value is 1, it indicates a perfect classifier.


(7) $$AUC=\frac{\sum {\left(p_{i},n_{j}\right)}_{p_{i}>n_{j}}}{P+N}$$

Where *P* is the positive sample number, *N* is the negative sample number, *p_i_* is the positive sample prediction score and *n_j_* is the negative sample prediction score.

The mean value of the above five indicators is recorded as the *score*, which serves as an evaluation index for the model’s average performance. To validate the effectiveness and stability of our machine learning and deep learning models, we conducted systematic sampling of the test set at varying proportions, ranging from 10% to 100%. For each proportion, we conducted 10 random samplings and calculated the average value as the result for that proportion. We used the Student’s t-test to perform statistical analysis on the performance parameters under 10 rounds of different testing strategies to validate the effective improvement of model performance.

### 2.5 Visualization and quantification of high-dimensional data

Uniform manifold approximation and projection (UMAP) (37) was employed as a dimensionality reduction technique to visualize high-dimensional data. This allows for the visualization of the distribution characteristics of such data. Analyzing the feature state within a neural network can contribute to enhancing the interpretability of the results. The downscaled features were visualized by means of a joint plot from the Python Seaborn library, which incorporated scatterplots and kernel density estimation plots.

To quantitatively evaluate the distribution of features, we utilized the silhouette score (38), a metric designed for assessing clustering quality in order to characterize the distribution of the extracted features. The value of the silhouette score ranges from [-1, 1]. The closer the value is to 1, the better the clustering effect; conversely, the closer it is to -1, the poorer the clustering effect.

## 3 Results

In this section, we first visualized the raw data distribution of samples from two systems and presented the statistical results of photoacoustic spectrum characterization. The results of ablation studies, where the model’s performance is tested following the removal of key design features, are demonstrated on the photoacoustic spectrums. Additionally, a comparison between traditional photoacoustic spectrum characterization and the proposed deep learning models was carried out. The final results showcase the architecture’s capacity to enhance the robustness of photoacoustic features and improve the diagnosis performance.

### 3.1 Raw data visualization and characterization

**Figure 3:**
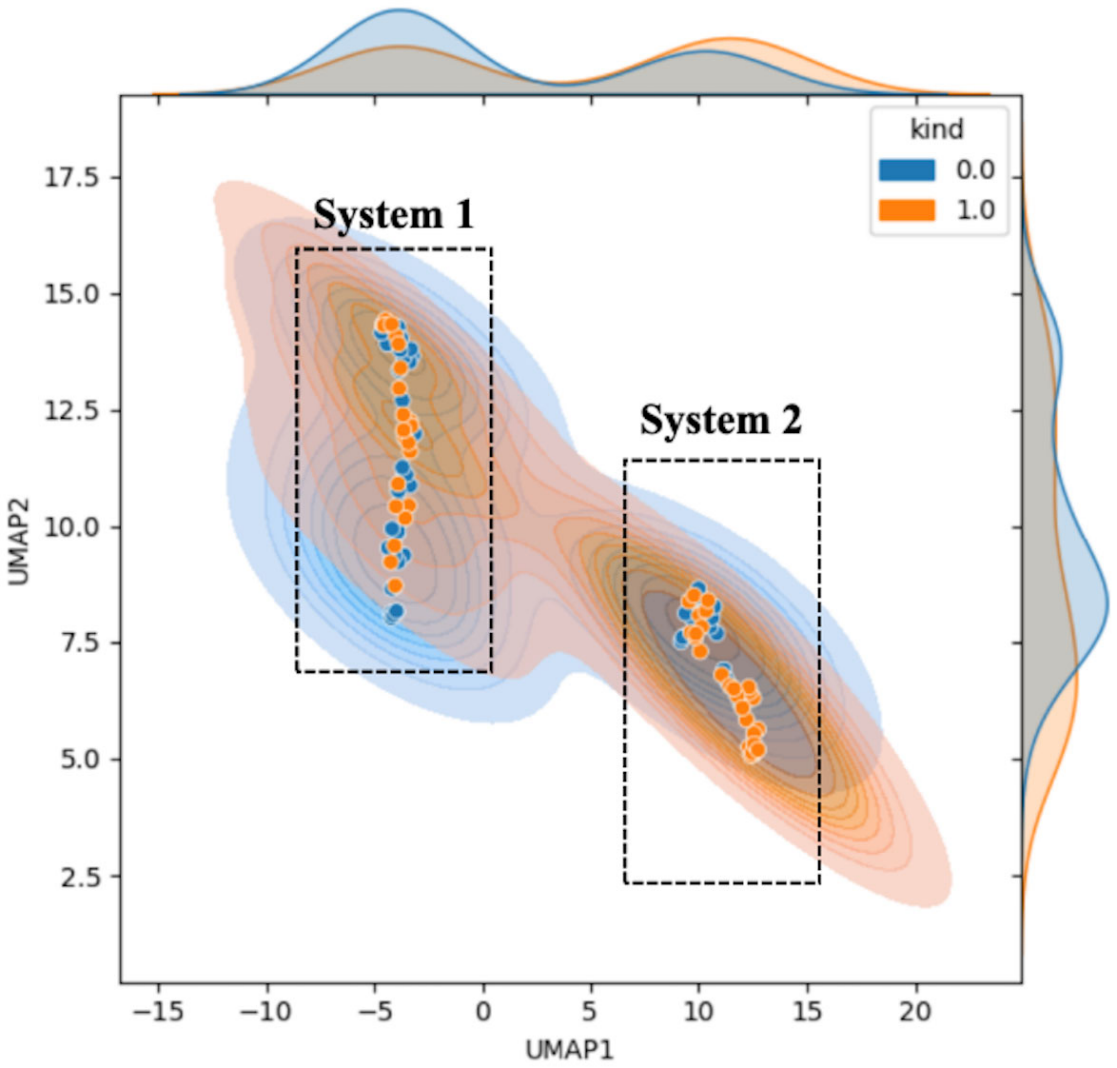
Feature visualization plot of the original sample data. The horizontal and vertical coordinates represent the UMAP dimensionality-reduced covariates. Blue indicates normal samples labeled as 0, while orange represents tumor samples labeled as 1. The figure includes the scatter plot (solid dots) of the samples, the sample probability distributions (semi-transparent distribution region in the figure), and the nuclear probability curves (separate density curves on the marginal axes).

**Figure 3** shows the results of the downscaled visualization of raw photoacoustic spectrum from both two systems using UMAP. The joint plot displays signal samples acquired at various point locations, along with separated kernel density distribution curves at the upper and right edges. The two black dotted frames respectively contain the sample data collected from two systems. System 1 employs the energy of the blackbody as a means of calibrating the laser energy. Conversely, system 2 resorts to the energy profile of the outgoing field for the calibration procedure. It is clear from the figure that there is a significant difference between the two sets of data. This is quite consistent with our conventional impression that different collection systems will cause deviations in sample distribution. And this will obviously have an impact on the extracted features.

**Figure 4:**
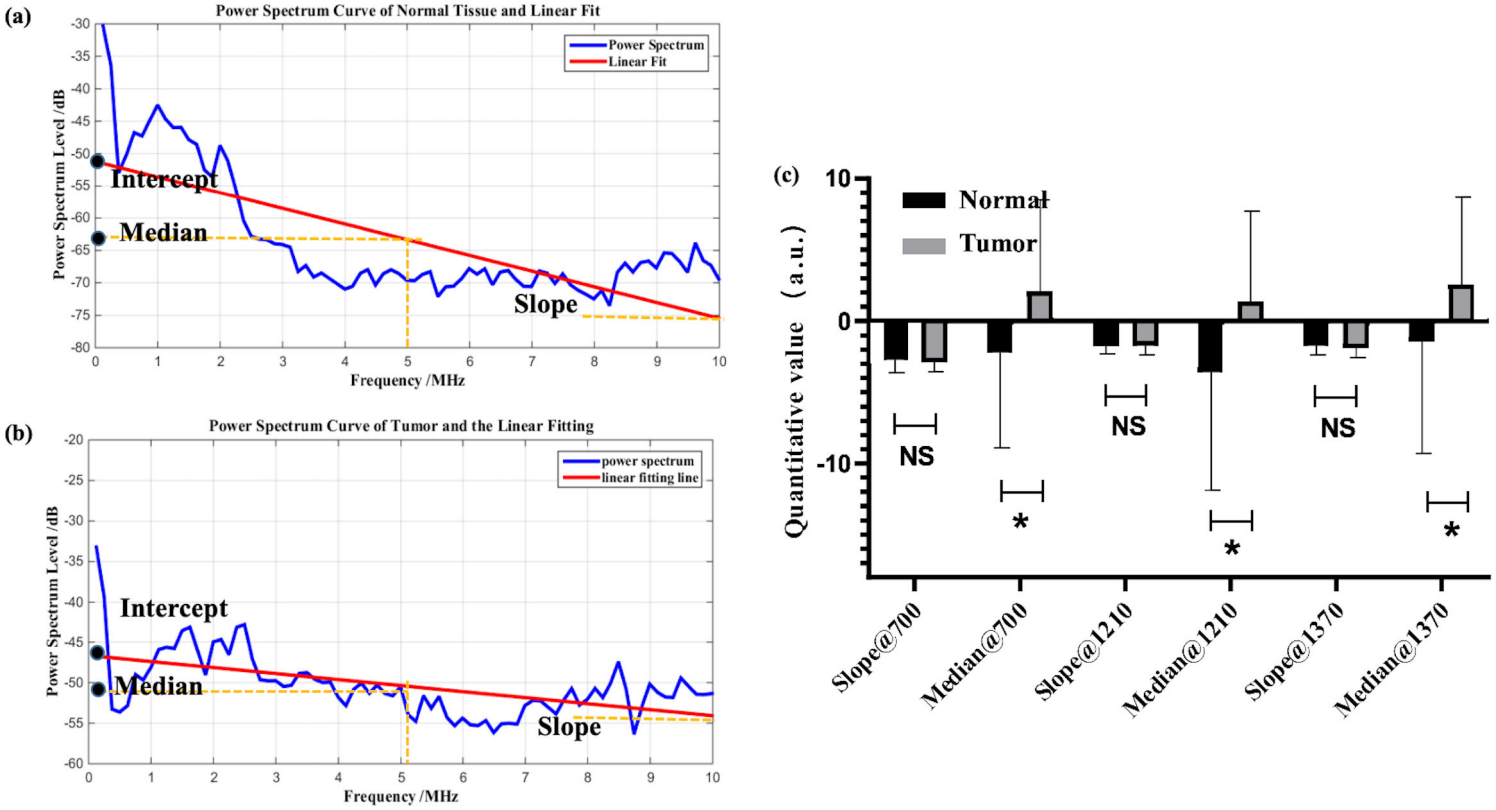
Example of linear fitting of photoacoustic spectra for normal **(a)** and tumor **(b)** samples; **(c)** statistical analysis of characteristic parameters, where * denotes 0.01<p value<0.05, NS is no significance.

**Figure 4** is the characterization result of photoacoustic signals, including the statistical analysis of the slope and the median at three different characteristic wavelengths. Based on clinical fundamentals and our preliminary research, prostate cancer exhibits enhanced structural heterogeneity compared to normal tissues, which is reflected in photoacoustic power spectrum as changes in the proportion of high and low frequencies. This can be quantitatively characterized by the slope of the first-order linear fitting of the photoacoustic power spectrum. From **Figures 4a, b**, the feature that the slope of normal samples is smaller than that of tumor samples is still retained. This is consistent with previous studies. However, statistically, the difference between the two could not be demonstrated (**Figure 4c**). The primary reason is that for the entire tissue, the photoacoustic signal represents an average of the signals within the irradiated area, which obscures the tissue heterogeneity. Thus, according to actual statistical results, the statistical effect is weakened.

In **Figure 4c**, the median shows statistical significance. Although this statistical result supports the median as a classification index for prostate cancer, in reality, combining with previous research foundations, the median is not a very suitable indicator. During tumor evolution, changes in the content of specific chemical components such as collagen and lipids can be partially reflected by the intercept or median of the power spectrum’s first-order linear fitting. In particular, the median is less affected by outliers compared to the intercept. However, the median only represents the signal intensity at the central frequency and may not necessarily align with the actual characteristic dimensions of the tissue. Additionally, as an intensity parameter, it is also vulnerable to noise and individual variability, which can be observed from the fact that the median exhibits greater variance than the slope in **Figure 4c**. Moreover, in this study, statistical results show that the median parameter of tumor samples is larger than that of normal samples. However, clinical guidance indicates that the content of collagen and lipids decreases in prostate cancer, which may be an error caused by individual variability and sample limitations. Therefore, although the median is statistically distinguishable for prostate cancer, its physical meaning is unclear and it is easily influenced by other factors.

### 3.2 Ablation study

An ablation study was conducted to determine the degree to which each design feature of the described model is necessary for the accurate photoacoustic diagnosis of the prostate cancer. After removing or changing from the model what we considered to be the key design features, the model was retrained on data with same random parameter initializations (random seed was set to 42). **Table 5** summarizes the results. When the designed SCL model was removed as part of the ablation study, all other features remained unchanged from the original architecture. Besides, the number of the convolution layers and the kernel dimension were also compared to verify the model validation. **Table 5** indicates the change of model in each experiment and reports the corresponding quantitative classification performance. Each model was trained to convergence using the same training set as described in Section 2D. Though the AUC of the model with one convolution layer and 2-D kernel is a little bit higher, it remains poor precision and recall. It can be happened in disease diagnosis indicating an occasion that the method can almost correctly identify people without the disease as being disease-free (high specificity). However, it will only diagnose people as being ill when their symptoms are very obvious or their condition is very severe (low sensitivity). When the specificity is high, it means that the false positive rate is very low in the ROC (Receiver Operating Characteristic) curve. Even if the sensitivity is low, as long as the curve can show a certain upward trend in the true positive rate on the basis of a low false positive rate, the AUC (Area Under the Curve) may be relatively large.

**Table 5:** Performance obtained when testing on variant model architecture.

Architecture Variant	ACC	Precision	Recall	Specificity	AUC	Score
2 Conv. layers, 2-D kernel w/ SCL (proposed)	0.81	0.89	0.68	0.88	0.91	0.85
2 Conv. Layers, 2-D kernel w/o SCL	0.71	0.86	0.54	0.88	0.90	0.78
1 Conv. Layers, 2-D kernel w/o SCL	0.33	0	0	0.88	0.94	0.43
3 Conv. layers, 2-D kernel w/o SCL	0.59	0.79	0.37	0.88	0.80	0.69
2 Conv. layers, 1-D kernel w/o SCL	0.58	0.67	0.55	0.50	0.52	0.56

**Figure 5:**
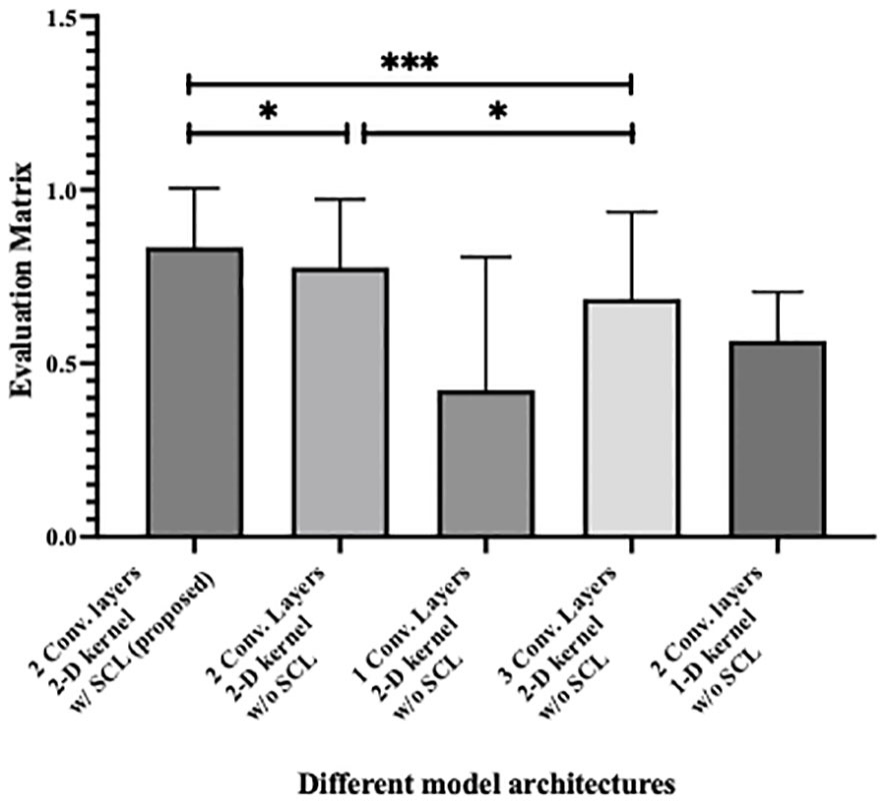
Statistical analysis of performance between different model architecture, where * denotes 0.01<p value<0.05 and *** denotes p value<0.001.

**Figure 5** shows the statistical analysis of performance between models, where parameters from 10 rounds of different testing strategies were all treated as variables for p-value calculation. It can be seen from the figure that the 2-layer CNN model based on 2D convolution, with or without the integration of SCL loss, demonstrates better performance. Additionally, compared with the relatively high-performing 3-layer CNN, both show effective performance improvements, among which the model with SCL loss added exhibits a more significant enhancement.

### 3.3 Experimental validation

The proposed deep learning model was further validated by utilizing the experimentally measured PA spectrums of two systems, as detailed in Section 2A. With reference to Section 2D for data partitioning and training, the training hyperparameters of each model after random parameter selection are shown in **Table 6**. Its performance was then compared with that of photoacoustic spectrum characterization methods and machine learning based methods. An additional comparison with the SC model was also carried out.

**Table 6:** Training hyperparameters of different models.

Training hyperparameters	CNN	SC model	SCL-adjust model
LR	0.001	0.01	0.01
MOM	0.7	0.95	0.95
Ratio	/	/	0.7

**Table 7** summarizes the quantitative results. As shown in the table, the SCL-adjust model outperforms other methods in most metrics. The model demonstrates high discriminant accuracy (ACC) and precision. It also exhibits a lower missed diagnosis rate (recall) and better model stability (AUC) compared to other models. Although the Specificity metric of the SCL-adjust model is slightly lower than that of LDA across all methods – indicating a slightly higher likelihood of false positives – false positives are more tolerable than high false negatives when photoacoustics is employed as a non-invasive primary screening system. It can be observed that conventional signal-processing based methods possess lower accuracy. This can be substantiated by **Figure 4**, where the difference in parameters is not highly significant and there is a large variance. Machine learning based methods encompass linear discriminant analysis (LDA) and quadratic discriminant analysis (QDA), as we have utilized previously in reference (17).

**Table 7:** Comparison with other methods.

Different methods	Paras or Methods	ACC	Precision	Recall	Specificity	AUC (with 95% CI)
Conventional signal-processing based methods	Slope@700	0.60	0.67	0.40	0.80	0.60 [0.45, 0.73]
	Mediam@700	0.50	0.67	0.33	0.75	0.65 [0.50, 0.76]
	Slope@1210	0.40	1.00	0.33	1.00	0.57 [0.41, 0.69]
	Mediam@1210	0.50	0.67	0.33	0.75	0.65 [0.50, 0.77]
	Slope@1370	0.40	1.00	0.33	1.00	0.61 [0.45, 0.74]
	Mediam@1370	0.50	1.00	0.38	1.00	0.62 [0.47, 0.74]
Machine learning based methods	LDA	0.64	0.66	0.42	0.90	0.70 [0.56,0.85]
	QDA	0.72	0.72	0.55	0.83	0.78 [0.65, 0.91]
Deep learning-based methods	CNN	0.71	0.86	0.54	0.88	0.90 [0.76 1.00]
	SC model	0.72	0.87	0.56	0.88	0.91 [0.72, 1.00]
	SCL-adjust (proposed)	0.81	0.89	0.69	0.88	0.91 [0.78,1.00]

**Figure 6:**
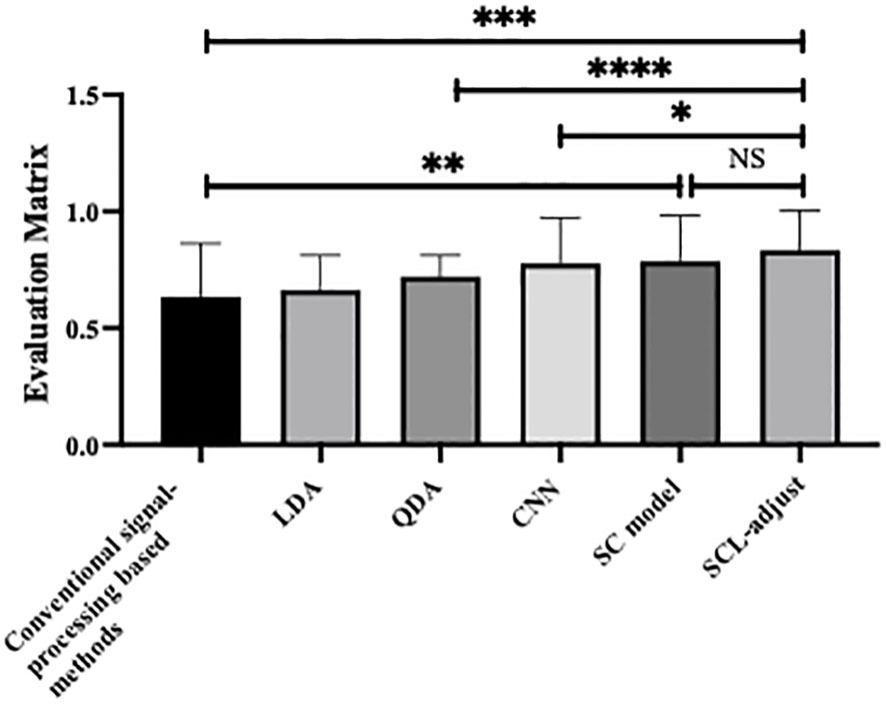
Statistical analysis of performance of different method, where * denotes 0.01<p value<0.05, ** denotes 0.001<p value<0.01, *** denotes p value<0.001 and NS is no significance.

**Figure 6** is the statistic results of all methods. As shown in the figure, deep learning demonstrates stronger feature representation capabilities compared to traditional methods and machine learning approaches, leading to significant improvements in performance across all metrics. Although the performance enhancements among the three deep learning-based models are not highly pronounced, the SCL model overall outperforms the other two models in all performance indicators, and its improvements are more significant than those of the other two methods.

**Figure 7:**
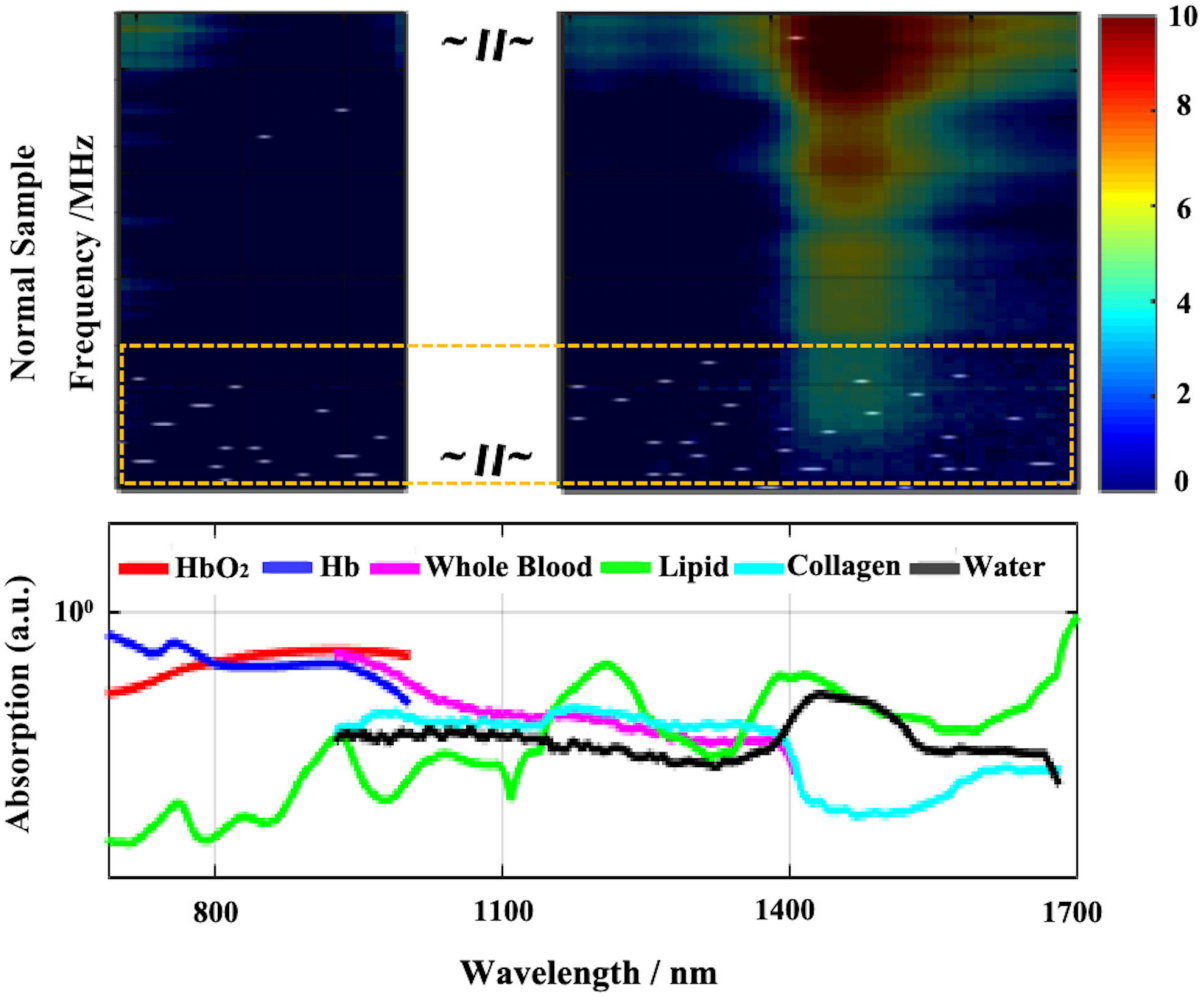
Visualization of LDA screened features (white dots in the above part, most of them are in the yellow dotted box). The above is the superposition result of the feature map and the original photoacoustic spectrum, and the below is the absorption curve of different components.

When LDA is used for binary classification, the feature can only be one-dimensional. According to the prior work, enhanced results can be achieved following feature screening and subsequent combination discrimination. After conducting feature screening using the validation set, we overlay the selected features of LDA onto the photoacoustic spectrum for visualization and is shown in **Figure 7**. The white points in the figure are the feature points extracted based on the validation set. Compared with the absorption spectrum, the white points are scattered across the entire characteristic spectrum. Whether in the absorption bands of hemoglobin or the characteristic bands of collagen and lipids, these features all influence the judgment of LDA. Intriguingly, the majority of the screening features are situated at high frequencies, which harbor more heterogeneous information.

Deep learning methods demonstrate an enhancement in prediction performance, particularly for the proposed SCL - adjusted model. To assess the robustness of the models, uniform and Gaussian noise were independently incorporated into the test set. To amplify the effect, the amount of noise data was augmented to ten times that of the sample data. To observe the noise resistance ability of the model on the test set, we exclude the samples from the test set that were incorrectly predicted and solely analyze the impact of noise on the samples that were originally predicted accurately.

**Figure 8:**
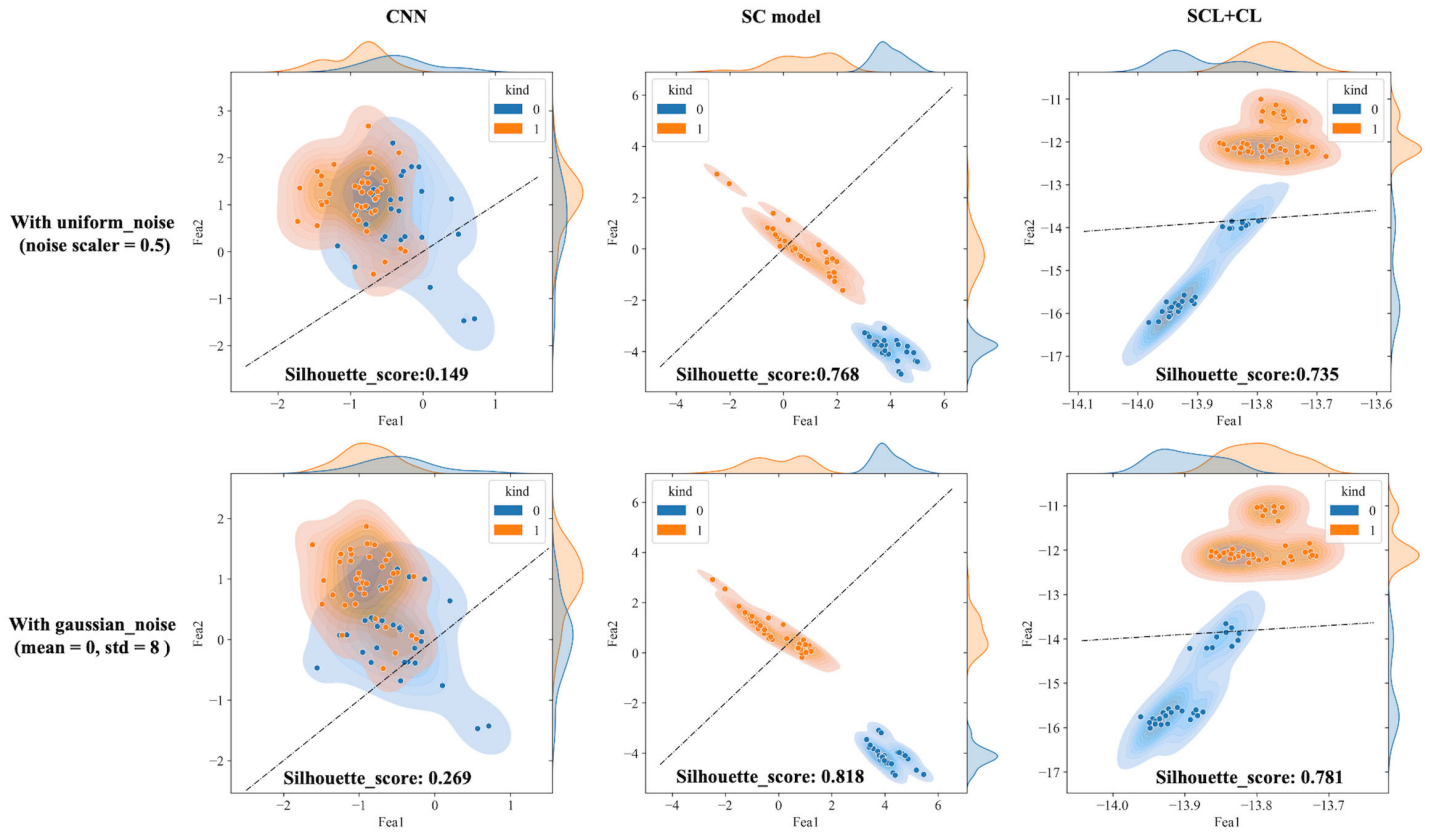
Visualization of three deep learning models extracted features after adding noise (black dotted line is the discriminant limit).

**Figure 9:**
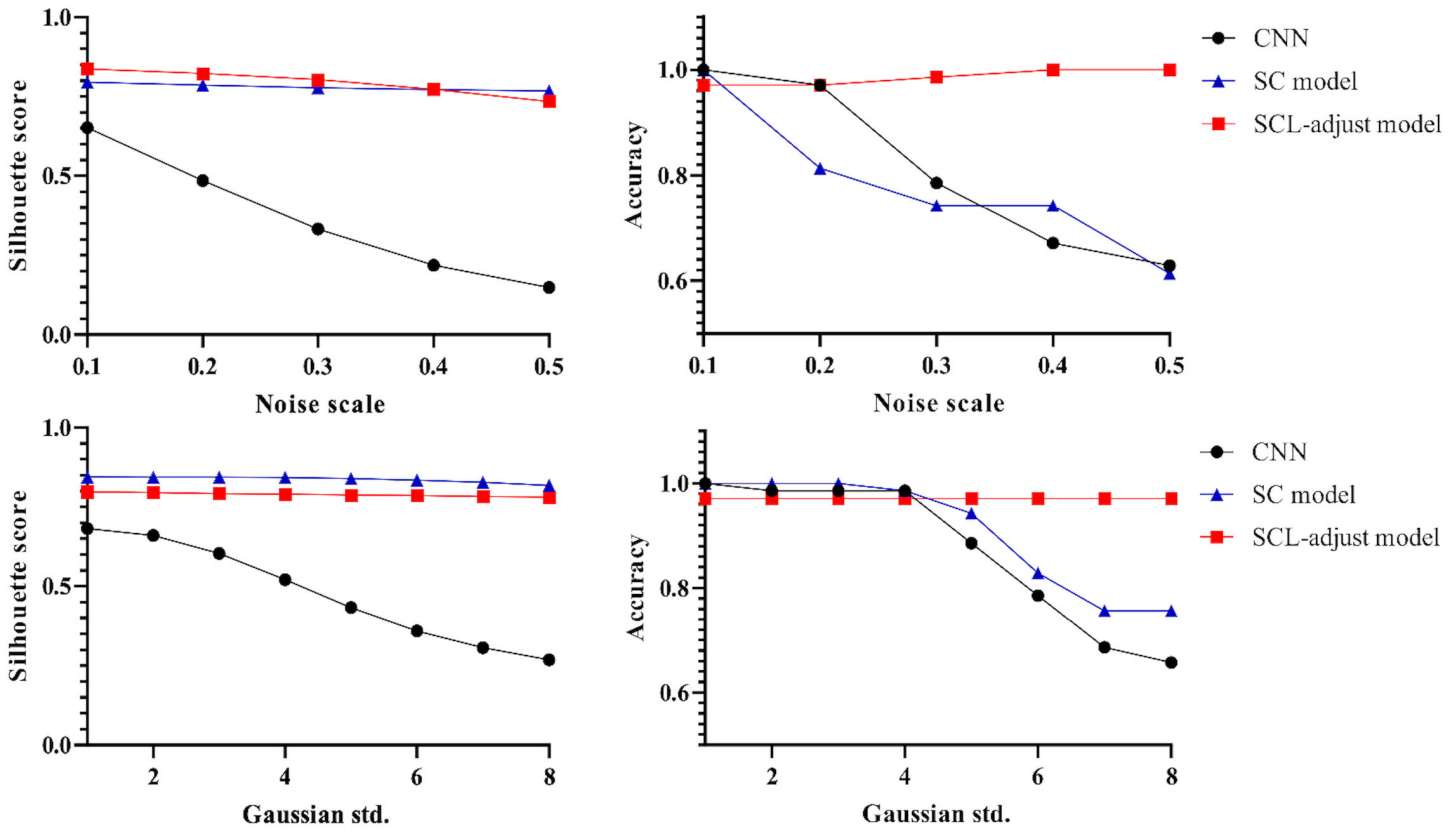
Influence of different noise parameters on feature distribution and discrimination accuracy.

The outputs of the model’s final layer before cross-entropy calculation are visualized t for a more in - depth analysis, as presented in **Figure 8**, and silhouette scores are calculated. Since the models are for binary classification tasks, the final layer outputs a 1×2 vector, with the two values in the vector corresponding to the x-axis and y-axis in the figures. For the two-dimensional output, the model selects the label corresponding to the larger feature value as the predicted value. Based on this, the line y=x is described as the decision boundary. Owing to the variation in the range of the image coordinate axes, a certain visual disparity may exist. Nevertheless, it can be discerned from the silhouette scores that the scores of the two contrastive learning models are higher than those of the CNN model. This indicates that the contrastive loss exerts an influence on the distribution of features, thereby enhancing the clustering and separation effect. As can be observed from **Figure 8**, following the addition of noise, the sample dispersion of the CNN model is remarkably increased, and a substantial number of samples exhibit classification errors. The silhouette score also remains at a relatively low level. Interestingly, the SC model has the highest silhouette score but is significantly affected by noise. Perhaps it is precisely due to the aggregating capability of contrast learning for features within the class that it is prone to discrimination errors of related samples when the sample is perturbed. Different noise parameters were analyzed and are presented in **Figure 9**. It is demonstrated that the SCL - adjust model performs better in both uniform and Gaussian noise. Consequently, the SCL - adjust model combines the strengths of the CNN model and supervised contrastive learning and is regarded as the optimal model for photoacoustic spectrum analysis in prostate cancer classification.

**Figure 10:**
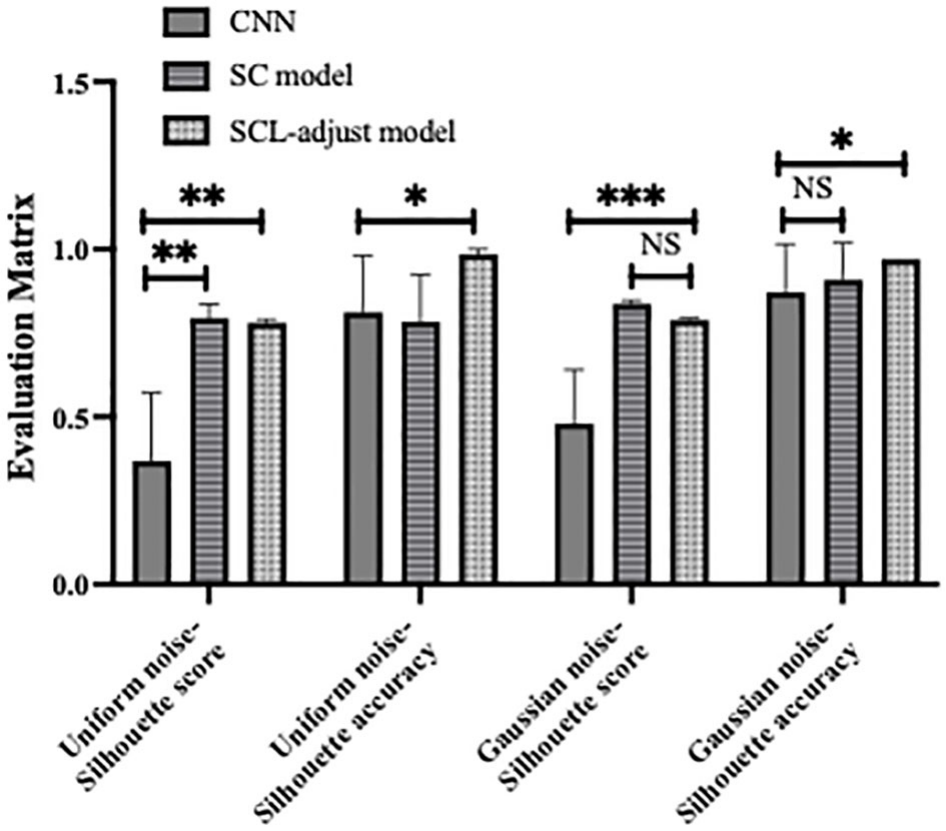
Statistical results of model performance metrics under different noise conditions, where * denotes 0.01<p value<0.05, ** denotes 0.001<p value<0.01, *** denotes p value<0.001 and NS is no significance.

**Figure 10** shows the statistical analysis results of multiple noise experiments in **Figure 9**, from which results largely consistent with the above can be obtained. The integration of contrastive loss, whether in the SC model or the SCL-adjust model, optimizes feature distribution compared to CNN, as reflected by the higher silhouette scores of the two models. This indicates closer intra-class distances and farther inter-class distances. Although the average silhouette score of the SC model is slightly higher than that of the SCL-adjust model, there is no significant difference between the two. It is precisely due to this characteristic that the SCL-adjust model maintains better performance in the presence of noise. The reason why the accuracy of the SC model does not remain as stable as that of the SCL-adjust model is likely because training the feature extractor and classifier independently makes it difficult to balance the two when the data volume is limited. The SCL-adjust model compensates for this issue by superimposing loss functions, making it a more suitable solution for small-sample scenarios.

**Figure 11:**
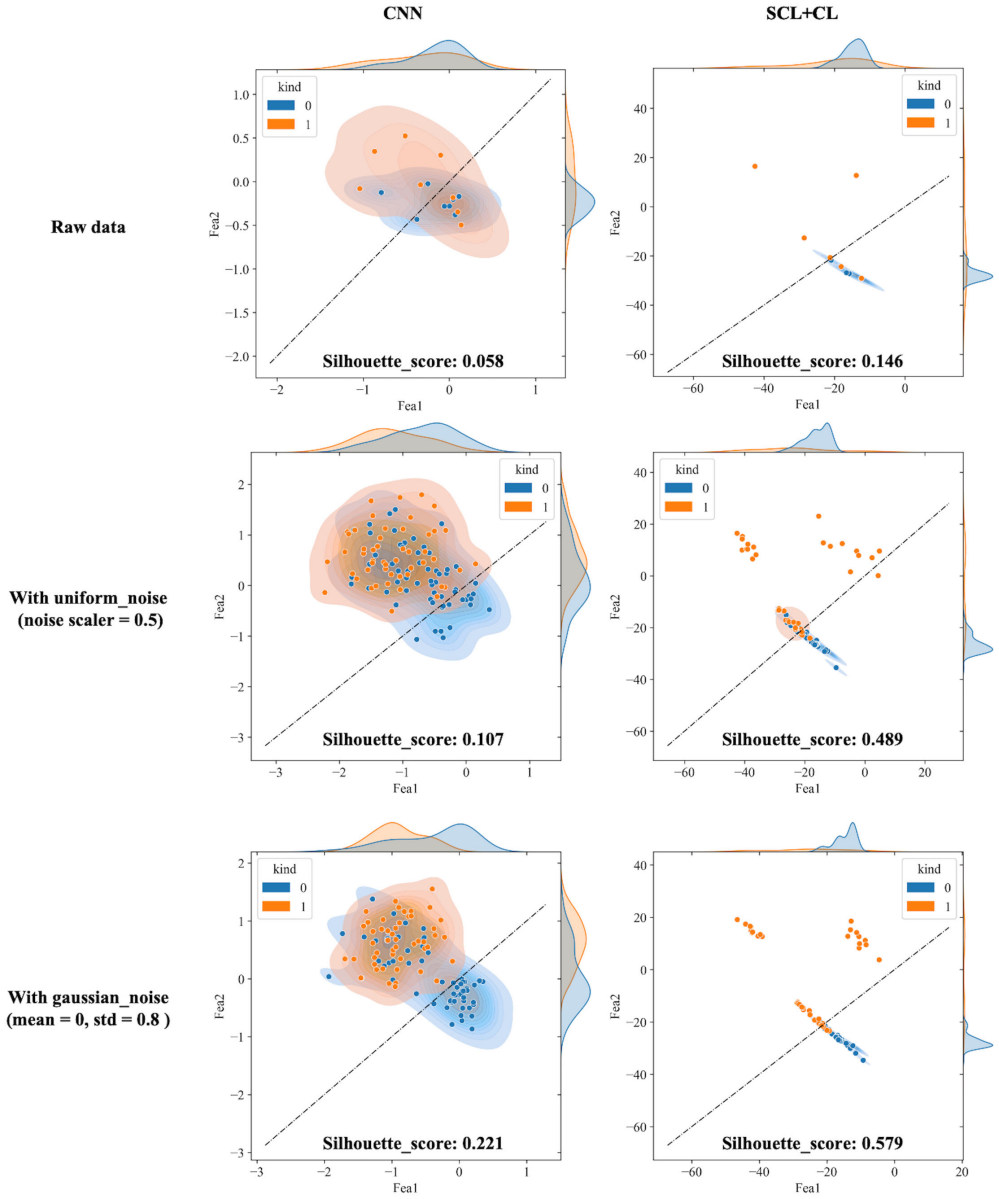
Visualization of three deep learning models extracted features after adding noise (black dotted line is the discriminant limit).

**Figure 12:**
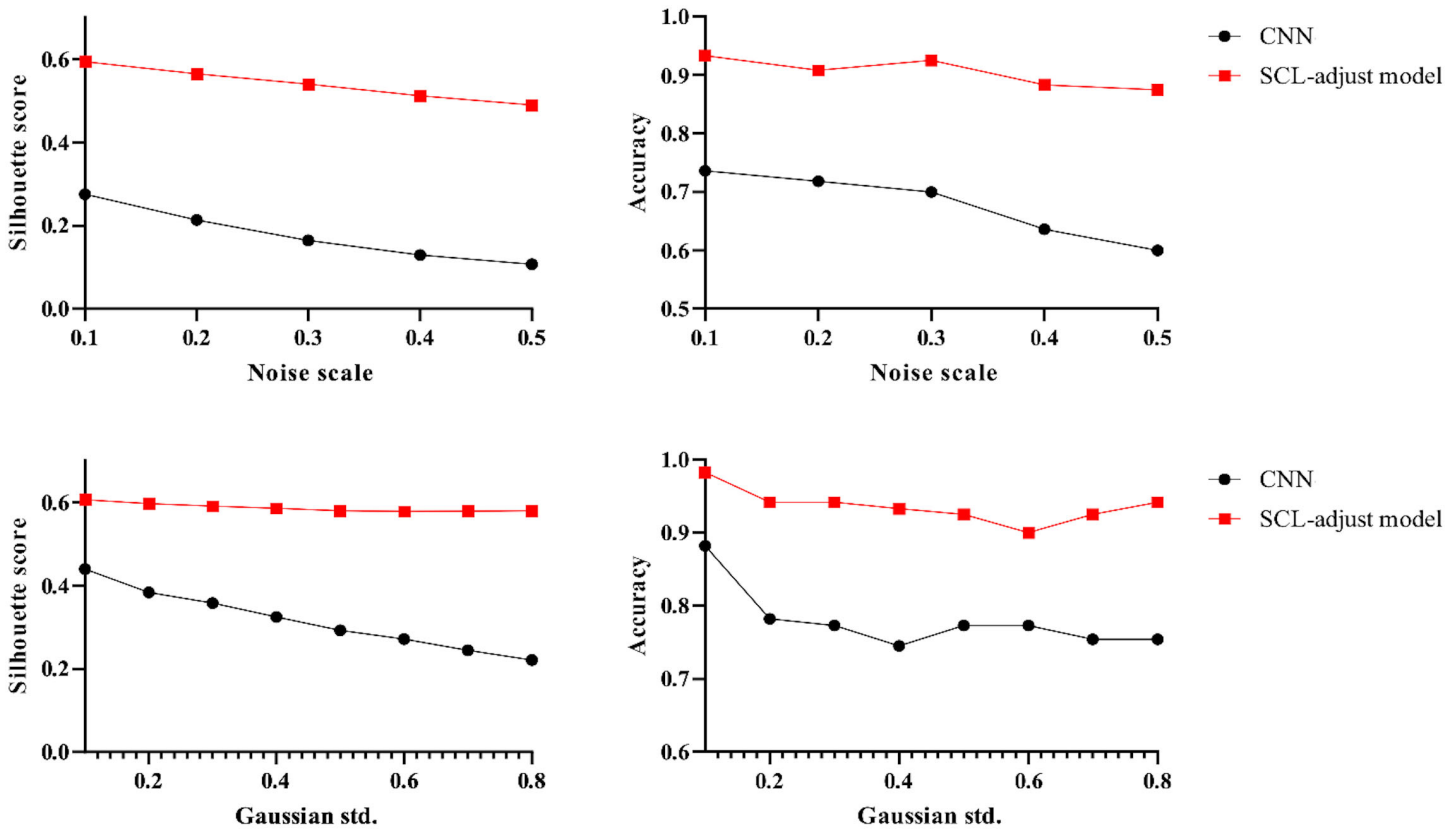
Influence of different noise parameters on feature distribution and discrimination accuracy.

**Figure 13:**
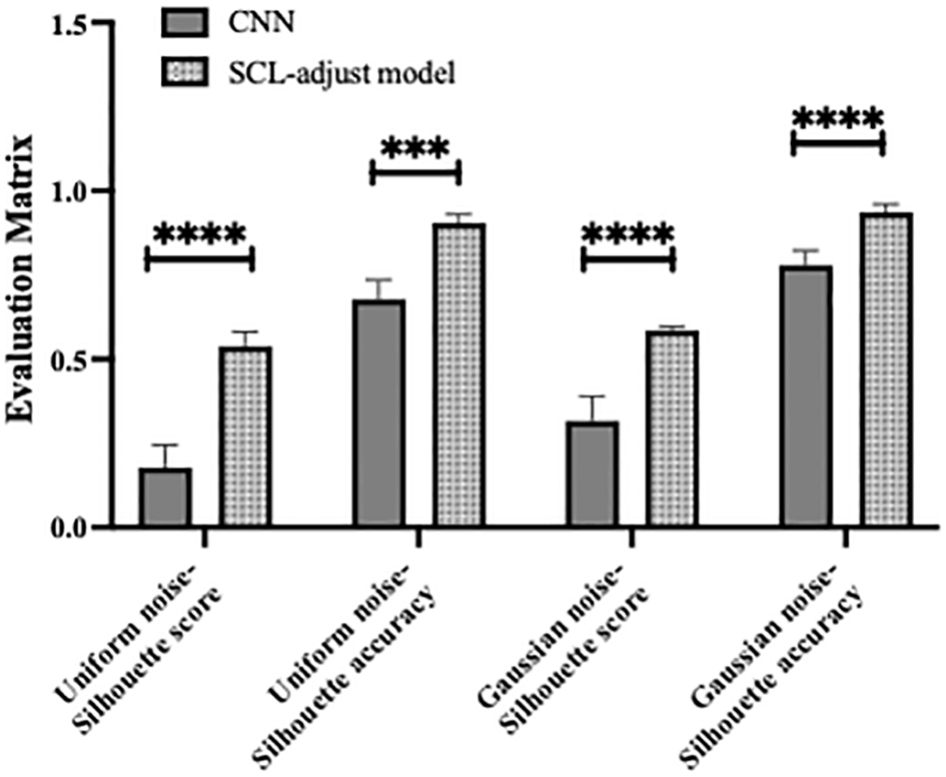
Statistical results of model performance metrics under different noise conditions, where *** denotes p value<0.001 and **** denotes p value<0.0001.

The proposed SCL-adjust model has also been verified using data from the other system. In the preliminary experiments, the classification performance of the SC model did not demonstrate a significant improvement compared to the CNN. Moreover, its model complexity and training difficulty were considerably higher than those of the CNN. Consequently, we no longer take this model into account and only compare the CNN with the proposed model. The discriminant effect of the SCL-adjust model remains superior. **Figures 11** to **13** present the corresponding noise influence results. The processing is identical to that of the previous dataset. By comparing the two datasets from different systems, the same conclusion can be drawn, indicating that the SCL-adjust model is robust across different datasets. We conducted a comparison of the models’ scores on two datasets from the two systems in order to assess which model exhibits superior performance in diverse situations. The results are presented in **Table 8**. When the models were transferred to the other system, the performance of both models deteriorated. This is understandable as it was caused by the variations in data distribution. Evidently, the proposed SCL-adjust model maintained better performance, both in terms of classification accuracy and noise resistance.

**Table 8:** Comparison of the models’ scores on two datasets from two systems.

Models	System 1	System 2	Transfer decline
CNN	0.826	0.696	15.7%
SCL+CL	0.882	0.790	10.4%

**Table 9** shows the number of parameters and other computational metrics of three models, including Floating Point Operations (FLOPs), memory usage, the time required for one training epoch, and the time required to test all data.

**Table 9:** The computational complexity of different models.

Models	Parameters (M)	FLOPs (M)	Memory usage (MB)	Training time per epoch (s)	Testing time (s)
CNN	0.20	138.1	22.65	0.05	0.02
SC model	0.76	140.0	30.50	0.14	0.03
SCL-adjust model	0.20	138.1	22.65	0.12	0.02

As can be seen from the table, the SC model requires independent training of the encoder and predictor using SCL (Supervised Contrastive Learning) and CEL (Cross-Entropy Loss) respectively, resulting in higher parameter count and computational consumption than the other two models. The SCL-adjust model adopts the same architectural framework as the CNN, so its parameter count and computational load remain consistent with the CNN. However, due to the integration of SCL in the backpropagation process, its single-epoch training duration increases significantly. With the increase in sample size and batch size, the computational consumption of the SCL-adjust model will rise. The three trained models share identical architectures; hence their testing times are relatively close.

## 4 Discussion

CNN is believed to have the ability to extract the spatial patterns of high-dimensional data. Through training and testing on the experimentally collected data, the CNN model learns to extract the necessary features for classifying prostate cancer. Owing to the preciousness of samples, the limited sample size restricts the depth of the neural network. Although the architecture and hyperparameters have been optimized via ablation study and random search (**Table 5**), it exhibits low resistance to noise and poor adaptation to data heterogeneity (**Figures 7**-**10**). Supervised contrastive learning is incorporated as an additional strategic design to enhance performance. As anticipated, the model incorporating contrastive concepts tends to yield more accurate predictions of prostate cancer.

Initially, a contrastive learning model (SC model) was trained and tested. We observe that the SC model has better feature clustering (**Figure 7**), yet it sacrifices classification accuracy (**Figure 8**). We hypothesize that it is precisely due to the aggregating capability of contrast learning for features within the class that it is prone to discrimination errors of related samples when the sample is perturbed. Therefore, the SCL-adjust model is developed to balance SCL and CE. The ratio evidently impacts the performance; thus, we search for the ratio from 0.1 to 0.9 with an increment of 0.1 and determine the optimal ratio of 0.3, and the results demonstrate better noise resistance and classification performance.

In this research, we only introduce noise during the evaluation process because our intention is merely to assess the noise resistance of different models, rather than to train the model to disregard the noise. Since our objective is to reduce sample heterogeneity, and training with samples from different individuals has achieved this goal, that is, the model has learned the principle to overlook sample heterogeneity. Adding noise to the testing dataset can magnify data heterogeneity as well as diversity, thereby further validating the robustness of the model. Additionally, we note that the difference lies in the fact that the Gaussian noise parameters of the second dataset are smaller. This is mainly because the normalization objects of the two sets of data are different, and the energy of the blackbody is much lower than that of the laser energy profile. Consequently, the magnitudes of the two sets of data vary, leading to different Gaussian noise parameters. The addition of uniform noise is determined by the product of the signal amplitude and the ratio, so the uniform noise parameters of the two sets of data are consistent.

The results reveal a transfer decline on the datasets from two systems. Although we use the term “transfer,” this is not transfer learning in the strict sense. Here, we trained the model on two datasets separately to verify the validity of the model with different datasets. It is evidently not convenient enough for reliable application, but it remains a common strategy currently that the model is retrained for use with another dataset. Transfer learning is considered a more favorable strategy for different datasets, whether through model fine-tuning or feature-domain adaptation. This is also our next step of work.

Data were collected from two distinct systems, with one equipped with a blackbody calibration block and the other lacking such a component. These two represent commonly employed methods for laser energy disrupt calibration. Despite the sole difference lying in the energy calibration approach, a pronounced disparity in data distribution was observed. When taking into account various photoacoustic systems, additional factors come into play, such as differences in hydrophone response and diverse experimental parameters, all of which have the potential to impact the diagnosis outcome. In this article, we verified the robustness of the contrastive loss in the representation feature, and this aspect can be further explored and analyzed with a broader range of data sourced from different systems, thereby facilitating a more comprehensive understanding and potentially uncovering novel insights.

Photoacoustic spectrum represents a typical high-dimensional data. The difficulty in sample collection also gives rise to the issue of small sample sizes. Although over 100 sample points may seem like a substantial accumulation, when compared with the feature dimension, it can still be regarded as a small sample problem in high-dimensional space. This article solely centers around model architecture and parameter control strategies for the purpose of preventing overfitting and guaranteeing that its accuracy is on a par with that of MRI. Feature reduction and sample augmentation are two prevalent strategies for handling small samples in high dimensions. By integrating photoacoustic data characteristics or leveraging unsupervised auto-encoders, selecting appropriate preprocessing techniques to eliminate redundant features or augment the data are potential avenues for further bolstering data consistency and elevating discrimination accuracy. Multispectral photoacoustic spectra can also be regarded as multi-modal data, and multimodal analysis can present novel perspectives for high-dimensional data analysis of photoacoustic spectra.

## 5 Conclusions

In this paper, supervised contrast learning is incorporated into photoacoustic spectrum analysis. Moreover, a comprehensive analysis is conducted on the performance improvement of the proposed SCL-adjust model in photoacoustic prostate cancer diagnosis, its resistance to noise, and its adaptability to the data heterogeneity of different systems.

The experimental results demonstrate that the feature distribution can be optimized either by incorporating supervised contrast loss into the loss function (SCL-adjust model) or through two-stage training (SC model). Via the comparison of noise disturbance, the proposed SCL-adjust model strikes a balance between the advantages of SCL and CEL and exhibits enhanced anti-noise capabilities. The proposed model is validated using data from two systems, yielding comparable outcomes, which attests to its proficiency in robust feature extraction compared to a single CNN model. In comparison with other methods, the proposed model generally outperforms in various indicators.

## Data Availability

The original contributions presented in the study are included in the article/supplementary material. Further inquiries can be directed to the corresponding author/s.
